# Non-destructive pre-symptomatic detection of gray mold infection in kiwifruit using hyperspectral data and chemometrics

**DOI:** 10.1186/s13007-023-01032-y

**Published:** 2023-06-02

**Authors:** Najmeh Haghbin, Adel Bakhshipour, Hemad Zareiforoush, Sedigheh Mousanejad

**Affiliations:** 1 Department of Biosystems Engineering, Faculty of Agricultural Sciences, University of Guilan, Rasht, Iran; 2 Department of Plant Protection, Faculty of Agricultural Sciences, University of Guilan, Rasht, Iran

**Keywords:** Early detection, Fungal infection, Hyperspectral imaging, Kiwifruit, Wavelength selection

## Abstract

Application of hyperspectral imaging (HSI) and data analysis algorithms was investigated for early and non-destructive detection of *Botrytis cinerea* infection. Hyperspectral images were collected from laboratory-based contaminated and non-contaminated fruits at different day intervals. The spectral wavelengths of 450 nm to 900 nm were pretreated by applying moving window smoothing (MWS), standard normal variates (SNV), multiplicative scatter correction (MSC), Savitzky–Golay 1^st^ derivative, and Savitzky–Golay 2^nd^ derivative algorithms. In addition, three different wavelength selection algorithms, namely; competitive adaptive reweighted sampling (CARS), uninformative variable elimination (UVE), and successive projection algorithm (SPA), were executed on the spectra to invoke the most informative wavelengths. The linear discriminant analysis (LDA), developed with SNV-filtered spectral data, was the most accurate classifier to differentiate the contaminated and non-contaminated kiwifruits with accuracies of 96.67% and 96.00% in the cross-validation and evaluation stages, respectively. The system was able to detect infected samples before the appearance of disease symptoms. Results also showed that the gray-mold infection significantly influenced the kiwifruits’ firmness, soluble solid content (SSC), and titratable acidity (TA) attributes. Moreover, the Savitzky–Golay 1^st^ derivative-CARS-PLSR model obtained the highest prediction rate for kiwifruit firmness, SSC, and TA with the determination coefficient (R^2^) values of 0.9879, 0.9644, 0.9797, respectively, in calibration stage. The corresponding cross-validation R^2^ values were equal to 0.9722, 0.9317, 0.9500 for firmness, SSC, and TA, respectively. HSI and chemometric analysis demonstrated a high potential for rapid and non-destructive assessments of fungal-infected kiwifruits during storage.

## Introduction

Kiwifruit (*Actinidia deliciosa*) is a commercial horticultural product cropped in several countries, mainly China, New Zealand, Italy, Greece, and Iran [1]. Its desirable taste, and remarkable health benefits due to great nutritional and medicinal values, caused kiwifruit to become very popular worldwide, as it is called the king of fruits [2]. According to statistical data from the FAO [3], the worldwide production of kiwifruit reached 2.84 megatons in 2010 to about 4.46 megatons in 2021. Iran ranks fifth among the kiwifruit producers, with more than 0.29 megatons in 2021 [3]. An important issue regarding kiwifruit production and storage is that it is susceptible to fungal diseases that can lead to substantial crop storage losses [4]. *Botrytis cinerea* fungus is the most widespread and disturbing agent that causes postharvest decay in kiwifruit [5, 6]. More than 20% of spoilage in kiwifruit is due to the gray mold disease produced by *Botrytis cinerea* [7]. If not appropriately controlled, this decay can deteriorate about a third of the fruit [8]. Therefore, early diagnosis of *Botrytis cinerea* infection in kiwifruit is crucial so that appropriate measures can be taken to prevent severe crop deterioration and financial losses [9]. The *Botrytis cinrea* infection has a negative impact on the kiwifruit quality indices [10]. Three of these important quality indices are firmness, soluble solids content (SSC), and titratable acidity (TA). Firmness is a physical measure which depends on the internal cell structure of the fruit [11]. Firmness loss is an important indication for the end of the fruit's shelf life [12]. SSC and TA, which are highly correlated with the kiwifruit sweetness and acidity [13], have been reported as important metrics in kiwifruit taste and flavor evaluation [14, 15] and consumer acceptability [16]. Therefore, proper monitoring of these physiochemical parameters is very important for the effective control of kiwifruit postharvest storage.

Hyperspectral imaging (HSI) is a developing approach that incorporates spectroscopy and image-capturing technologies in one system to simultaneously obtain electromagnetic reflectance and pixel-wise information in a nondestructive and reliable way manner [17, 18]. Successful applications of HSI have been reported in several agri-food industry-related fields and summarized in recent valuable review articles [19–22]. In the case of fruits, HSI technique was used by Çetin et al. [23] for monitoring the qualitative attributes of apple at different harvesting times based on spectral reflectances at 386–1028 nm. The determination coefficient (R^2^) values were up to 0.910 for firmness prediction and 0.684 for SSC prediction, respectively. In another study, an accuracy of 99.4% was obtained by partial least squares-discriminant analysis (PLS-DA) for invisible damages in persimmon fruit [24]. There are also other studies related to the use of HSI in fruits, such as the identification of apple varieties [25], monitoring the postharvest variation of total flavonoids content (TFC) in Chinese dwarf cherry [26], maturity determination of okra pod [27], ripeness evaluation of bananito fruit [28], and kiwifruit [29]. The changes in the spectral reflectance signature of agricultural materials by fungal pathogens due to impacts such as fungi sporulation, depigmentation, lesion, necrosis, etc., can be used for disease monitoring [30]. Taking the advantage of pixel-wise spectrum imaging, the HSI technology has a high potential for early disease detection in agricultural products [31]. HSI has been applied for detecting fungal infection of fruits. This technology was used for differentiating healthy and early-molded blueberry fruits based on the influential spectral band of 685 nm to 1000 nm, in which the PLS-DA model discriminated the diseased blueberries with a rate of 99%. Liu et al. [32] conducted a study on identifying fungal diseases in peach fruit. The peach fruit samples were contaminated by three fungal pathogens of *Botrytis cinerea*, *Monilia fructicola*, and *Rhizopus stolonifer*. Hyperspectral data were collected at 400–1000 nm wavebands. They reported that the Principal Component Analysis (PCA) differentiated the infected samples into three different fungal infection level groups. Besides, the PLSR model predicted the fungal colony counts with R^2^ of more than 0.84. In another research article, an HSI system with 400 nm to 1000 nm spectral reflectances was employed by Jiang et al. [33] to distinguish three different degrees of natural mildew infection in *Camellia oleifera* fruit. The significant wavelengths by Competitive Adaptive Reweighted Sampling (CARS) algorithm were fed into the PLS-DA model, and a correct classification rate (CCR) of 83.3% was achieved. The HSI method was also used for detecting fungal infections in citrus fruit [34], stored apple fruit [35], and strawberry fruit [36]. Appropriate hyperspectral data analysis is fundamental to achieving the goal of proper model development. Some challenges that should be suitably addressed regarding hyperspectral images include sensor noises, high dimensions of the spectral dataset, and redundant information [37]. Chemometric algorithms are practical tools for handling and analyzing multivariate data provided in HSI systems [38] to decrease the computation time, enhance the model performance, and promote robustness by removing inappropriate and redundant variables [39]. These techniques are informatively described in a review article by Saha and Manickavasagan [40], some of which will be discussed and used in this manuscript.

Although the review of studies shows the feasibility of using the HSI to detect fungal diseases in some agricultural products, to the best of our knowledge, there is no report on the effectiveness of hyperspectral data for detecting and tracking the *Botrytis cinerea* contamination in kiwifruit. Therefore, this study aimed to investigate the capability of HSI combined with different data processing approaches and chemometrics for pre-symptomatic detection of moldy gray infection in kiwifruit. Data treatment and feature selection methods followed by modeling approaches were developed to track the disease and predict the variations of some qualitative indices in the Hayward kiwifruit due to the *Botrytis cinerea* fungal infection.

## Material and methods

### Sample preparation

In order to prepare the required samples, a total number of 225 fresh Hayward kiwifruits of almost the same size and without any defect or injury were picked carefully in an orchard near Fouman city, Guilan Province, Iran. The fruits were carefully cleaned with sterile water. In a random selecting manner, 15 batches of samples were provided, and each batch consisted of 15 kiwifruits. The first data collection was performed on the first day (called day zero in this study) on the kiwifruits of one batch. The kiwifruit samples of seven batches were inoculated with *Botrytis cinerea* pathogen, while no contamination was applied to the other seven batches. All the samples were maintained in a dark environment inside an incubator at 20 °C during the experiments. In order to prepare the seven batches of contaminated samples, a culture of *Botrytis cinerea* was obtained from the laboratory of the department of plant protection of the University of Guilan. The fungus spore contaminated the fruits of seven batches at the wound region made by removing the pedicle. The inoculation procedure was according to Liu et al. [5].

### Experimental measurements

Three important attributes in kiwifruit postharvest quality are TA, firmness, and SSC [41]. The firmness of fruits was determined using a portable penetrometer (FT 011, Effegi, Japan) with a tip radius of 8 mm, according to Ghasemnejad et al. [42]. Next, the SSC values of the juices of the samples (°Brix) were measured using a handheld refractometer (Euromex RD 635, Netherlands) [43]. Eventually, the juices of the kiwifruit samples were titrated with a 0.1 Normal Sodium hydroxide solution to measure the TA (% of citric acid) according to Asiche et al. [43]. Experiments were carried out on days 2, 5, 8, 11, 14, 17, and 20 based on the infection development and upon the advice of the plant pathology expert involved in this research. On the evaluation days, data was collected from one non-contaminated group and one contaminated group to consider the possible effects of fruit storage (e.g., fruit ripening) on the experiment results.

### Hyperspectral image acquisition

**Fig. 1:**
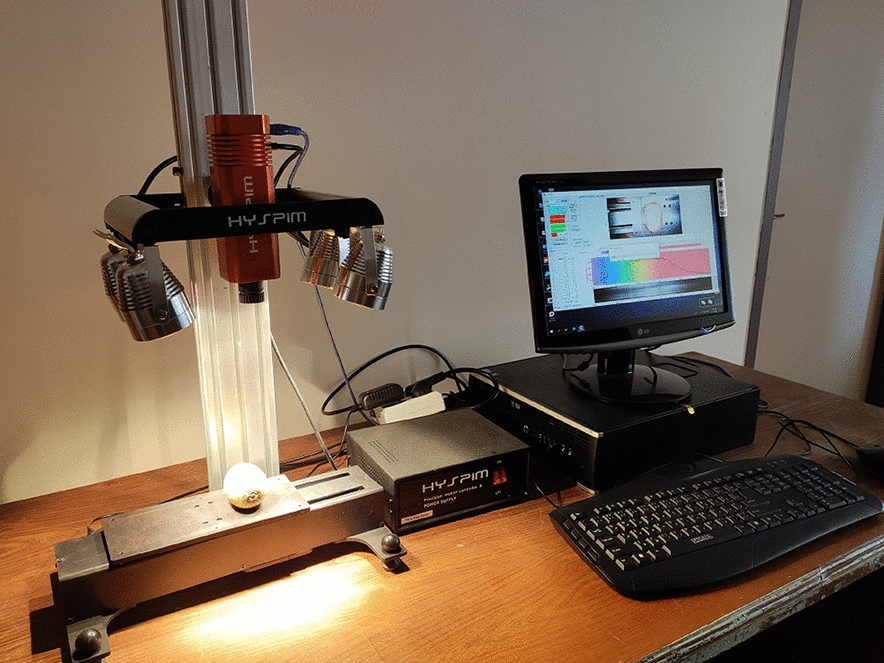
HSI setup used in this study

In this study a Vis–NIR HSI device (HYSPIM, Model HS_Vis-NIR-15fps, Iran) was used to obtain hyperspectral images with a wavelength band of 400–950 nm. The imaging system contained a line-scanning hyperspectral imager, four 150W halogen lamps installed symmetrically oriented at two sides of the imager for illumination, an RGB camera, a stepper motorized horizontally moving platform for sample placement, a control system, a computer processor, and a graphical user interface for hyperspectral image acquisition (Fig. 1). In order to capture the spectral responses, the single kiwifruit samples were placed on a black mate platform with a vertical distance of 25 cm from the lens of the imager. The exposure time was 0.3 s, and the platform velocity was 3 mm/s. The dimension of the obtained hypercube was 122 × n (depending on the scanning length) spatial pixels and 568 spectral bands. White and dark reference calibration was performed on the raw hyperspectral images using Eq. 1 [25, 44]:

1 $${R}_{calibrated}=\frac{{R}_{raw}-{R}_{dark}}{{R}_{white}-{R}_{dark}}$$

where ${R}_{raw}$ was the raw recorded hyperspectral image, ${R}_{calibrated}$ was the calibrated hyperspectral image, ${R}_{white}$ and ${R}_{dark}$ were the reference images, respectively. The dark reference image at zero reflectance was obtained when the lights were off, and an opaque cap was placed on the camera lens. The white reference image (reflectance of 99%) was obtained from the calibration whiteboard provided by the camera seller company. Finally, the calibrated data cubes were recorded using HYSPIM software graphical user interface (GUI) for further processing operations.

### Spectra preprocessing

In this study, five main actions, including spectral preprocessing, optimal spectral wavelength selection, model construction, and model performance evaluation, were sequentially carried out on the hyperspectral data to develop the classification and prediction models. The collected data cubes were loaded in MATLAB programming software (2021a, the MathWorks, USA). Initial evaluations showed high noise-to-signal ratio levels in the kiwifruit single-band images extracted from the both ends of the spectra. Therefore, through an initial screening, the wavelengths of 400–449 nm and 901.6–950 nm, which contained noticeable noises, were eliminated from the data cubes. Then, the wavelengths of 450–900.6 nm, including 465 bands, were selected from a total of 563 wavelengths for further analysis. Previous research also reported a similar approach [45, 46]. After removing highly noised spectral bands, three blocks of 4 × 4 pixels were cropped from the regions around the fruit pedicle point, where the infection had been applied. Finally, the average spectral values of these regions were calculated (per wavelength) to obtain the spectral reflectance curve of each sample.

### Hyperspectral data pretreatment

Pretreatment of the hyperspectral data is a crucial phase in spectral analysis that can reduce or eliminate the systematic errors caused by several environmental and instrumental issues [47]. In this study, five different pretreatment methods, including moving window smoothing (MWS), Savitzky–Golay 1^st^ derivative, Savitzky–Golay 2^nd^ derivative, multiplicative scatter correction (MSC), and standard normal variates (SNV) were applied to the extracted spectral data. MWS selects a window with a predefined size that moves on the spectrum and replaces the measured value of the wavelength points with the calculated average value at the central wavelength of the window [48]. MWS can decrease the local noises in hyperspectral data [49]. In this study, the spectral window size was set to five bands for the MWS filter. The SG algorithm is also a moving window approach, but instead of spectra average, a polynomial least squares fit of the spectra inside the window is calculated in this method [48]. The derivatives are calculated after applying the polynomial fitting [50]. The SG filtering with a window size of five points and a second degree polynomial was carried out in this study. MSC and SNV methods eliminate the undesired variations caused by light scattering without changing the curve of the original wavelength [50]. The main difference between MSC and SNV is that MSC uses data from all data sets to standardize the spectrums, while the SNV method uses the data of each particular spectrum to normalize that spectrum [51].

### Effective wavelength selection

The recorded information from the kiwifruit samples contained a large number of reflectance data in a wide wavelength range. Along with the useful information, these numbers of spectral features may contain redundant, non-informative, and multi-collinear information. Therefore, it is necessary to perform wavelength selection on the raw spectral data to remove the unuseful information and enhance the modeling efficiency and performance [52, 53]. In order to select the most informative wavelengths, three different variable selection methods namely uninformative variable elimination (UVE), competitive adaptive reweighted sampling (CARS), and successive projection algorithm (SPA) were applied to the spectral data. UVE selects the significant wavelengths by eliminating those which provide no or little information by setting a threshold on the partial least squares regression (PLSR) coefficients [54]. In this study, the significant UVE features were selected based on their reliability index (RI) method, which was explained by Wang et al. [55]. An artificial noise matrix with the same size as the spectra data was generated and added to the spectra matrix. The UVE was implemented on the data containing spectra and noise. After determining the noise data's absolute RI value, the wavelengths whose absolute RI value was less than the maximum absolute RI value of the noise matrix were eliminated. CARS performs wavelength selection by establishing PLS models on the N (N = 100 in this study) feature subsets derived based on Monte-Carlo (MC) sampling method. Then, the optimal variables combination is selected based on the lowest root mean square error of the model cross-validation [56]. The methodology of the CARS method is presented by Wang et al. [57]. In the SPA method, multiple linear regression (MLR) models are formed for several subsets of the wavelength vector, and the wavelengths with the least RMSE are designated as the most valuable wavelengths [56].

### Model establishment and evaluation

The selected characteristic wavelengths were fed into modeling tools to classify the fruits of different days after infection and predict the variations of quality attributes due to gray mold disease. LDA and SVM methods were used for discriminating the non-contaminated and contaminated kiwifruit samples on different days after *Botrytis cinerea* inoculation, while the PLSR and support vector regression (SVR) models were employed for predicting kiwifruit quality attributes. Model development was carried out in Unscrambler X software (version 10.4, CAMO ASA, Oslo, Norway). Different combinations of pretreatment, wavelength selection, and modeling algorithms were employed in each case for classification and prediction purposes. The total 225 samples were randomly splitted into two-third (150 samples) for calibration and one-third (75 samples) for evaluation. Ten-fold cross-validation method was used in the model calibration phase. The developed structures were compared and the most successful models were selected based on the statistical criteria. In the case of prediction, ${R}^{2}$ value of the calibration (${R}_{Cal}^{2}$), ${R}^{2}$ value of the cross-validation (${R}_{CV}^{2}$), ${R}^{2}$ value of the evaluation (${R}_{EV}^{2}$), RMSE of the calibration (${RMSE}_{Cal}$), RMSE of the cross-validation (${RMSE}_{CV}$), and RMSE of the evaluation (${RMSE}_{EV}$) were calculated. The models with the highest ${R}_{CV}^{2}$, and lowest ${RMSE}_{CV}$ values were the most desired. Therefore, the R^2^ and RMSE statistics were calculated using Eqs. (2) and (3) [58].

2 $${R}^{2}=1-\left[\frac{\sum_{i=1}^{N}{({y}_{exp,i}-{y}_{pred,i})}^{2}}{\sum_{i=1}^{N}{({y}_{exp,i}-\overline{{y }_{exp}} )}^{2}}\right]$$

3 $$RMSE={\left[\frac{1}{N}\sum_{i=1}^{N}{({y}_{exp,i}-{y}_{pred,i})}^{2}\right]}^{0.5}$$

where ${y}_{exp,i}$ and ${y}_{pred,i}$ were the *i*th experimental and predicted values, the N is the total samples. $\overline{{y }_{exp}}$ was the average of the experimental attribute. Moreover, the RMSE and accuracy metrics were defined to compare the classifiers. The accuracy parameter was determined using Eq. 4, in which TP, FP, TN, and FN were true positive, false positive, true negative, and false negative values extracted from the confusion matrices of classification models [59]. It should be stated that in order to include the effect of storage time in the analysis, all the non-contaminated samples were placed in one group as the control class.

4 $$accuracy=\frac{TP+TN}{TP+FP+TN+FN}\times 100$$

## Results and discussion

### Quality attributes

**Fig. 2:**
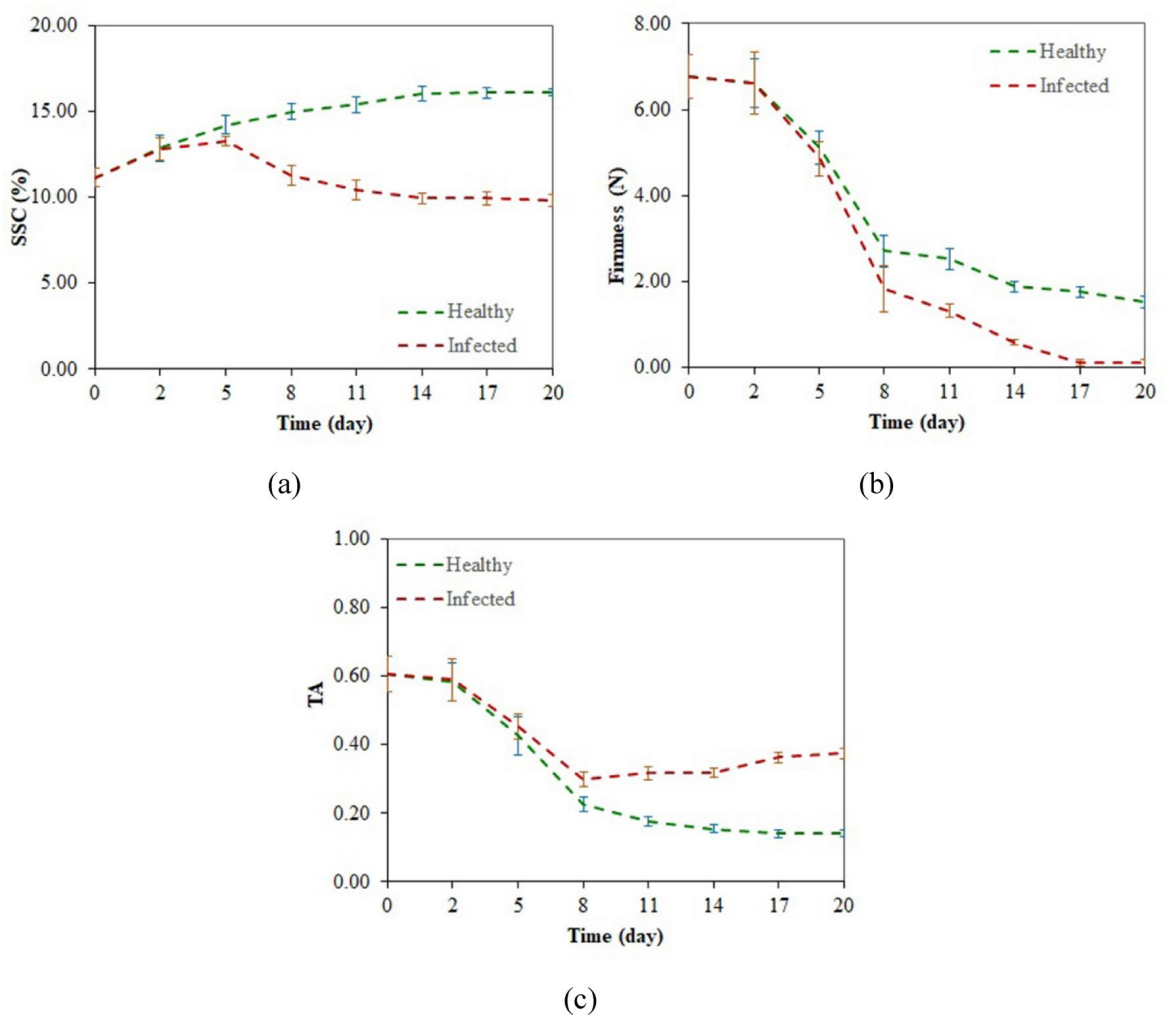
Variations in internal quality attributes of kiwifruit samples during the experiments after gray mold infection; **a** SSC, **b** firmness, and **c** TA

Figure 2 depicts the variations of kiwifruit internal quality attributes during the experiments after gray mold infection. It can be seen from Fig. 2a that the SSC value of the kiwifruit samples increased during the first five days and, while it continued to increase as time passed for non-contaminated samples, showed a downward trend for detected samples after day five. Since kiwifruit is a climacteric fruit, the changes in the internal characteristics of the non-contaminated fruits result from postharvest ripening, but for contaminated kiwifruit samples, the variations result from fruit ripening and the effect of disease development. It seems that the increase of SCC in non-contaminated samples and the initial increase in SCC of contaminated samples is due to the decomposition of starch into soluble sugars because of ripening [60, 61]. In contrast, after the first days, the development of the infection inside the contaminated kiwifruit samples decreases the glucose content and thus decreases SSC [62]. The decrease in fruit firmness (Fig. 2b) was predictable due to the ripening process and the destruction of fruit tissue caused by infection. A reduction in the TA value of samples was observed in non-contaminated kiwifruit samples during the days of the experiment. This downward trend during ripening is due to using organic acids for pyruvate decarboxylation [61]. Similar behavior was observed in contaminated fruits during the first days but followed by an increase in the latter days. Although the ripening process decreases the TA [43], the dominance of gray mold increases the citric acid content of the kiwifruit [62], increasing the TA index. It was also observed that the first apparent signs of *Botrytis cinerea* infection in kiwifruit samples appeared on the sixth day after inoculation. At the same time, monitoring of laboratory characteristics showed that the development of the disease was so high on the sixth day that it contaminated the quality attributes of diseased kiwifruit samples. Therefore, early detection of the disease before the appearance of symptoms is essential to prevent the deterioration of the infected fruits and the transmission of the disease to healthy fruits.

### Results of wavelength pretreatment

**Fig. 3:**
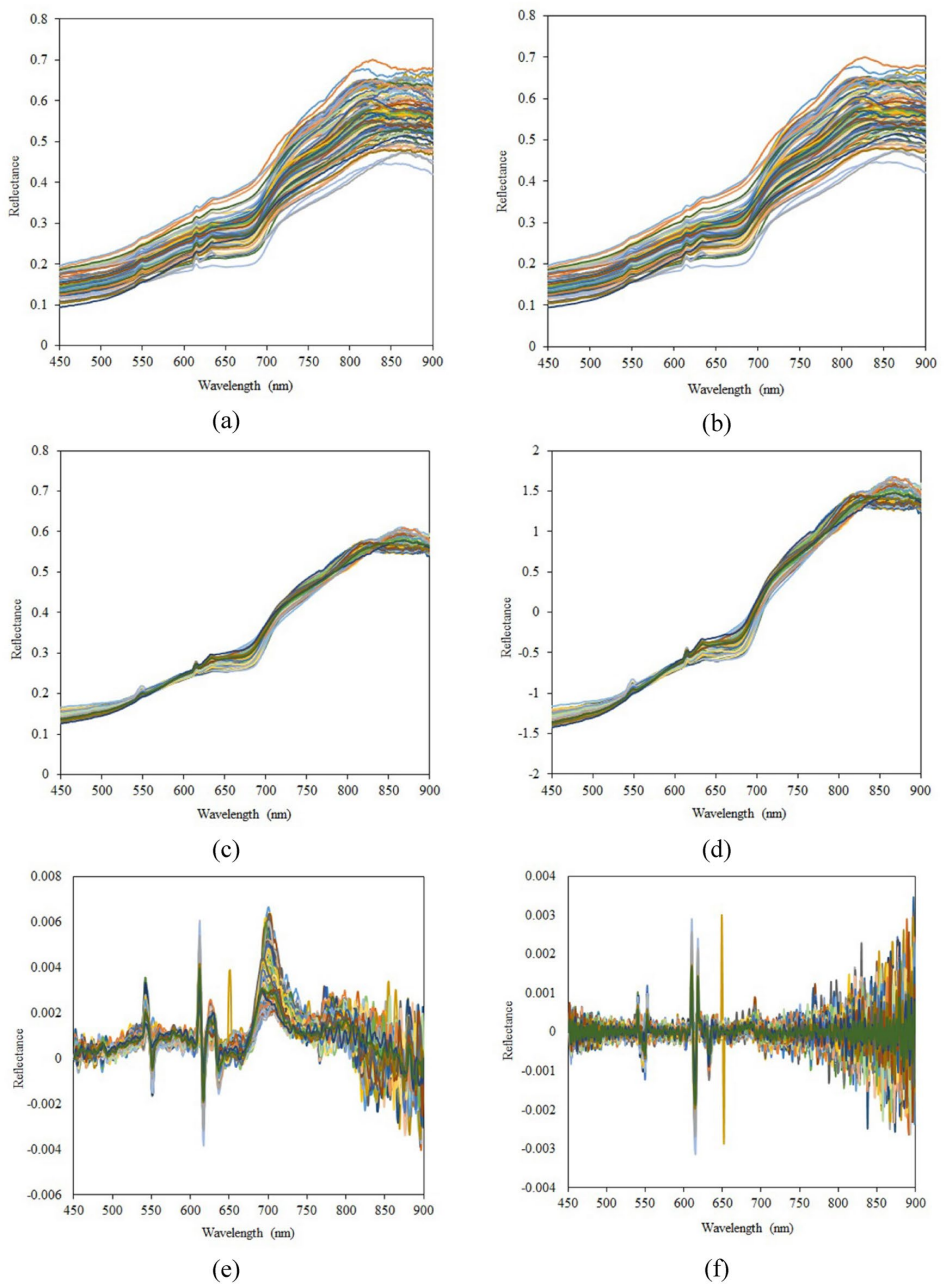
The spectra curves of raw (**a**) and preprocessed reflectance of kiwifruit samples using MWS (**b**), MSC (**c**), SNV (**d**), Savitzky–Golay 1^st^ derivative (**e**), and Savitzky–Golay 2^nd^ derivative (**f**) methods

Figure 3 shows the raw and preprocessed reflectance spectra of kiwifruit samples. Figure 3a shows the raw recorded spectral data after eliminating 400–450 nm and 900–950 nm wavelengths due to the high noise-to-signal rate. It can be observed that although the overall reflectance spectra of different samples have an almost similar trend, there are some differences in some wavelength ranges, which encourage further analysis of spectra curves. The reflectance values of different kiwifruit samples around the bandwidth regions of 600 nm to more than 700 nm and 800 to 850 nm show different variations.

Figure 3b shows that the mean filtering algorithm was able to partially reduce the local noises on the spectra curves. Still, the MWS-pretreated spectra were not meaningfully different from the raw data. The preprocessed reflectance spectra using MSC and SNV algorithms are presented in Fig. 3c and d, respectively. SNV and MSC can reduce those differences between the spectra which have been caused by particle size and scattering [63]. Considering an equal scale, the MSC-pretreated and SNV-pretreated reflectance curves were significantly closer than the raw data curves, which shows the effective de-noising performance of MSC and SNV algorithms in this study. Similar results were reported in previous studies on spectral-based food quality assessment [64–66]. Moreover, the fact that the overall trend of MWS, MSC, and SNV spectra remained similar to that of the original spectra shows that the collected spectra have the capability to monitor the actual properties of kiwifruit samples. Savitzky–Golay 1^st^ derivative and Savitzky–Golay 2^nd^ derivative spectra curves are depicted in Fig. 3e and f, respectively. It is observed that these pretreatment algorithms remove the baseline effects. There are several wavelengths with peak values in Savitzky–Golay 1^st^ derivative, which may contain characteristic information about the samples. The second derivative also removes the linear trend in the reflectance curve [67], which may lead to the loss of some information.

In order to select the most appropriate wavelength pretreatment methods for prediction goals to reduce the number of calculations, a pre-evaluation was carried out so that the raw data, as well as the pretreated data, were employed for model establishment before implementing any wavelength selection. In this case, PLSR was employed to develop the multivariate models. The corresponding results are shown in Table 1. It can be seen that among the evaluated spectra datasets, the Savitzky–Golay 1^st^ derivative gained the best performance criteria of ${R}_{Cal}^{2}$ of 0.9448, ${RMSE}_{Cal}$ of 0.5909, ${R}_{CV}^{2}$ of 0.9023, and ${RMSE}_{CV}$ of 0.7906 for predicting fruit firmness. The Savitzky–Golay 1^st^ derivative-PLSR model achieved the ${R}_{Cal}^{2}$, ${RMSE}_{Cal}$, ${R}_{CV}^{2}$, and ${RMSE}_{CV}$ of 0.9127, 0.3703, 0.7523, 0.6283, for SSC prediction, and 0.9594, 0.0232, 0.7841, and 0.0539 for TA prediction, respectively. Therefore, the Savitzky–Golay 1^st^ derivative-pretreated spectral values were selected as the optimal datasets for subsequent prediction analysis. Besides, the Linear Discriminant Analysis (LDA) models fed with different spectral data were evaluated to classify kiwifruit samples of the different days to select the best pretreatment method based on the accuracy and RMSE values (Table 2). These results show that the SNV algorithm was the best method with the ${accuracy}_{CV}$ of 93.33%, and the ${RMSE}_{CV}$ of 0.1291. Therefore, the SNV pretreated dataset was used for classification analysis.

**Table 1:** Results of PLSR models based on different pretreated spectral data

Target	Pretreatment	${R}_{Cal}^{2}$	${RMSE}_{Cal}$	${R}_{CV}^{2}$	${RMSE}_{CV}$
Firmness	None	0.9301	0.6650	0.8631	0.9402
	MWS	0.9336	0.6481	0.8916	0.8323
	MSC	0.9103	0.7531	0.8467	0.9953
	**Savitzky–Golay 1st derivative**	**0.9448**	**0.5909**	**0.9023**	**0.7906**
	Savitzky–Golay 2nd derivative	0.9226	0.6997	0.7469	1.2721
	SNV	0.9236	0.6950	0.8553	0.9673
SSC	None	0.7832	0.5836	0.6916	0.7028
	MWS	0.7182	0.6653	0.6455	0.7513
	MSC	0.8958	0.4046	0.7468	0.6368
	**Savitzky–Golay 1st derivative**	**0.9127**	**0.3703**	**0.7523**	**0.6283**
	Savitzky–Golay 2nd derivative	0.7317	0.6492	0.6180	0.7822
	SNV	0.8622	0.4652	0.7170	0.6736
TA	None	0.8197	0.0488	0.7329	0.0600
	MWS	0.8695	0.0415	0.7834	0.0540
	MSC	0.8446	0.0453	0.7400	0.0591
	**Savitzky–Golay 1st derivative**	**0.9594**	**0.0232**	**0.7841**	**0.0539**
	Savitzky–Golay 2nd derivative	0.8260	0.0480	0.5830	0.0750
	SNV	0.8211	0.0486	0.7321	0.0599

Bold text indicates the best pretreatment method

**Table 2:** Results of LDA classifiers based on different pretreated spectral data

Pretreatment	${accuracy}_{CV}$	${RMSE}_{CV}$
None	92.22	0.1359
MWS	90.00	0.1511
MSC	91.11	0.1424
Savitzky–Golay 1st derivative	90.00	0.1528
Savitzky–Golay 2nd derivative	73.33	0.2144
**SNV**	**93.33**	**0.1291**

Bold text indicates the best pretreatment method

It is also observed in Tables 1 and 2 that using some pretreated spectral data resulted in lower model performances in some cases. For example, using the MSC data improved the modeling performance compared to using raw data for SSC and TA. Meanwhile, the MSC data reduced performance for predicting firmness and classifying kiwifruit samples. This shows that applying some preprocessing methods for eliminating the noise and background scattering interference may cause the elimination of some information that may be potentially critical for some particular modeling purposes, resulting in lower distinguishing performances.

### Wavelength selection results

**Fig. 4:**
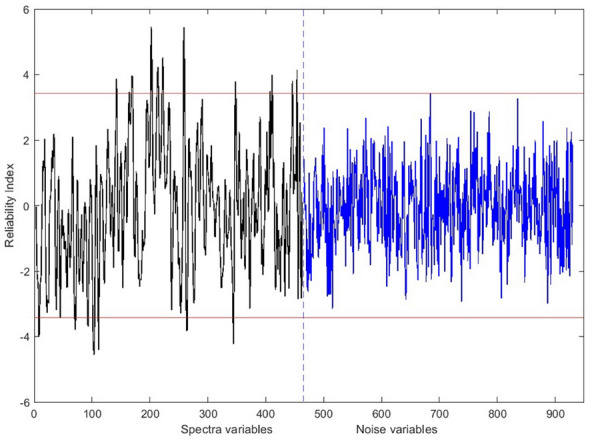
RI values of wavelengths and noise matrix resulted from applying UVE on the Savitzky–Golay 1^st^ derivative pretreated spectrum for predicting kiwifruit SSC

Based on the results of the previous section, the UVE, SPA, and CARS wavelength selection algorithms were applied to the Savitzky–Golay 1^st^ derivative spectra for prediction models. The mentioned algorithms were also applied to the SNV spectral data for classification models. Figure 4 shows the plot of the RI values determined by applying the UVE method on the Savitzky–Golay 1^s^^t^ derivative pretreated data for SSC prediction. The black graph shows the RI values of the wavelengths, and the blue part shows the RI values of the noise matrix. Red horizontal lines offer the range of RIs to be eliminated (± maximum absolute RI of noise matrix). There were 37 wavelengths selected for this item.

**Fig. 5:**
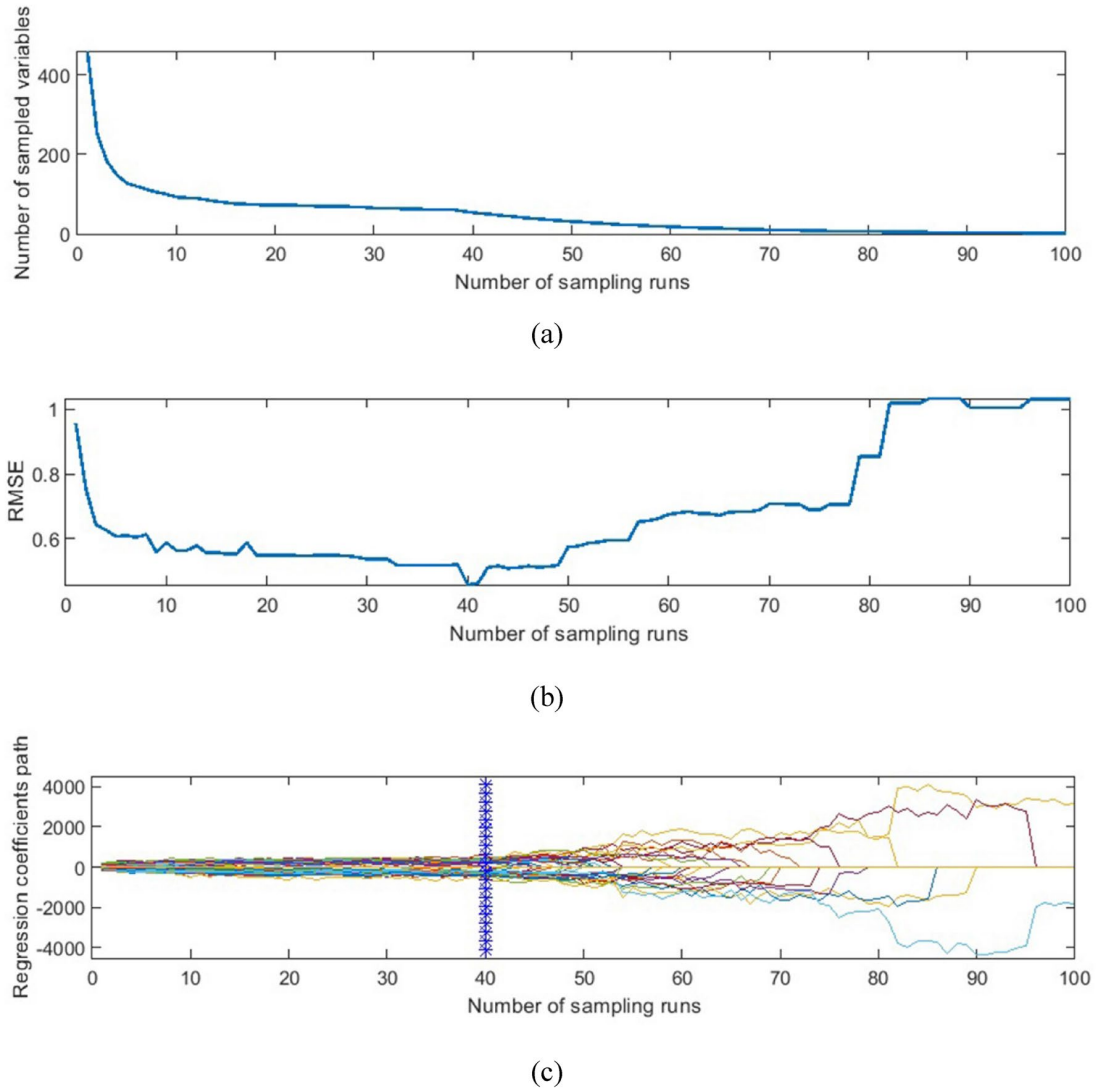
Result of applying CARS wavelength selection on the Savitzky–Golay 1^st^ derivative pretreated spectrum for predicting kiwifruit SSC, showing variation in the number of wavelengths (**a**), variation of RMSE (**b**), and the regression coefficient path (**c**) vs. the number of sampling runs

The results of applying the CARS algorithm on the Savitzky–Golay 1^st^ derivative pretreated data for SSC prediction are shown in Fig. 5. It is observed from Fig. 5a that the number of sampled wavelengths decreased rapidly at the initial step of MC sampling, which is called the fast selection phase. At the same time, the decreasing trend became much milder after the first sharp fall during the refined selection. This is due to the exponentially decreasing function (EDF). Refer to Yun et al. [68] and Li et al. [69] for good descriptions of EDF in feature selection. Variations of the RMSE value of tenfold cross-validation are depicted in Fig. 5b. The RMSE value decreased quickly at the first sampling runs, followed by a slight downward trend until the sampling run of 40 where the RMSE increased again. The vertical star line in Fig. 5c shows the optimal number of wavelengths which was 31 from 465 wavelengths (about 6.67%). The CARS algorithm was also reported as an effective wavelength selection method for the non-destructive prediction of Feicheng peach firmness during in-field ripeness [70].

**Fig. 6:**
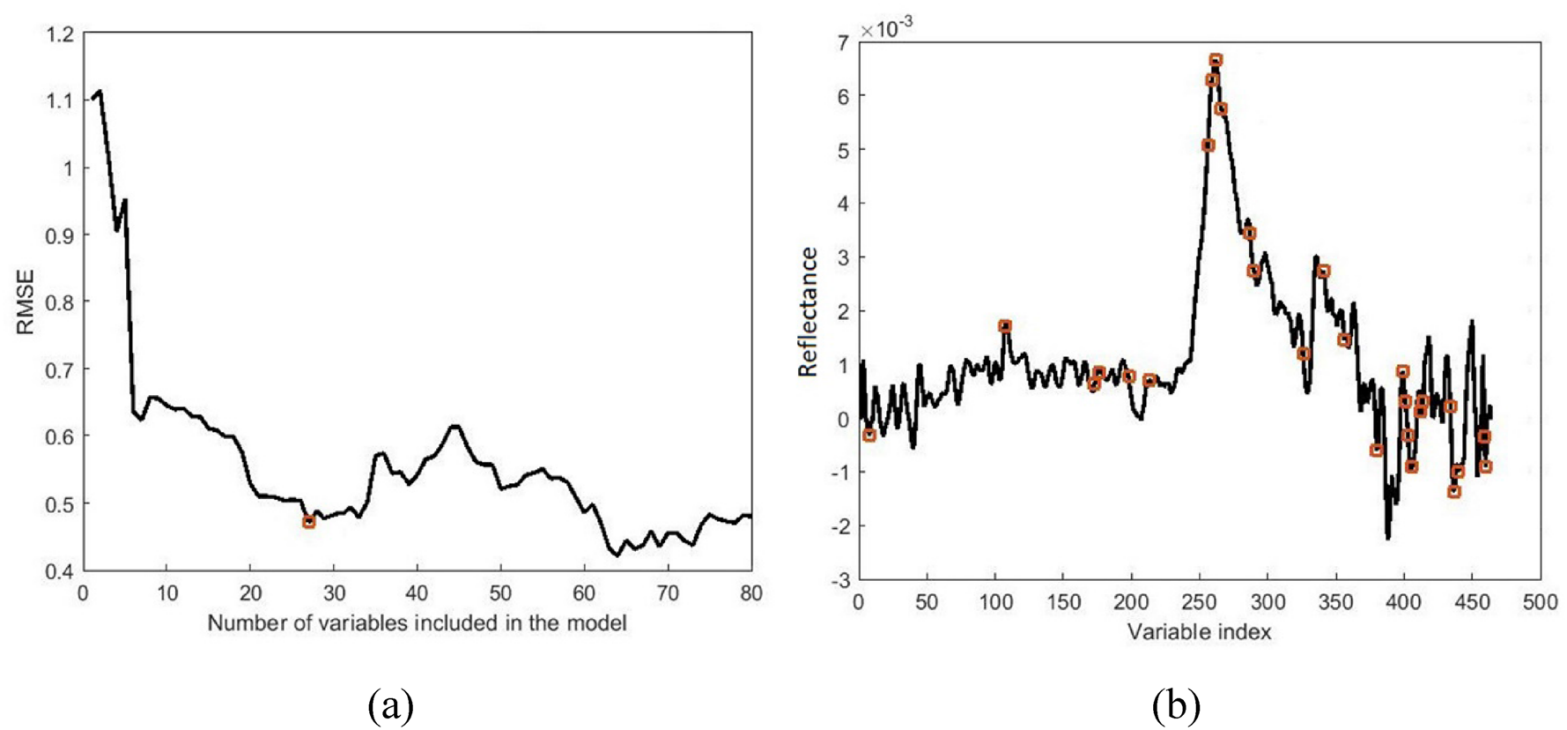
Result of applying SPA wavelength selection on the MWS pretreated spectrum for predicting kiwifruit SSC, showing the variation of RMSE vs. the number of wavelengths (**a**), and the selected wavelengths (**b**)

Figure 6 presents the results of SPA for wavelength selection in the case of SSC prediction using Savitzky–Golay 1^st^ derivative pretreated data. The variation of RMSE by changing the number of wavelengths is shown in Fig. 6a. It is observed that, after a slight increase for the initial two wavelengths, the RMSE of the SPA algorithm decreased by increasing the number of included variables. This decreasing trend continued until the number of included wavelengths reached 27, where again, a trivial increase was observed. Therefore, SPA extracted 27 valid wavelengths associated with kiwifruit SSC. Figure 6b shows the selected SPA-selected wavelengths marked on the spectrum. The number of wavelengths selected by CARS, SPA, and UVE algorithms for different purposes in this study is listed in Table 3. It should be noted that although the lower number of features is more desired for a simpler model, taking the accuracy of the models into account, sometimes applying more number of wavelength features may result a more accurate model. Furthermore, it is observed from Table 3 that the wavelength selection algorithms decreased the number of model input data to less than 10% of the original dataset. The SPA algorithm was applied successfully by Shao et al. [71] on the visible-infrared spectral wavelengths for quantitative assessment of three different tomato varieties.

**Table 3:** The number of selected wavelengths by different wavelength selection algorithms for classification and prediction models

Wavelength selection	Classification	Firmness prediction	SSC prediction	TA prediction
UVE	47	28	37	40
CARS	19	46	31	49
SPA	41	34	27	19

### Discrimination of non-contaminated and contaminated kiwifruits

**Fig. 7:**
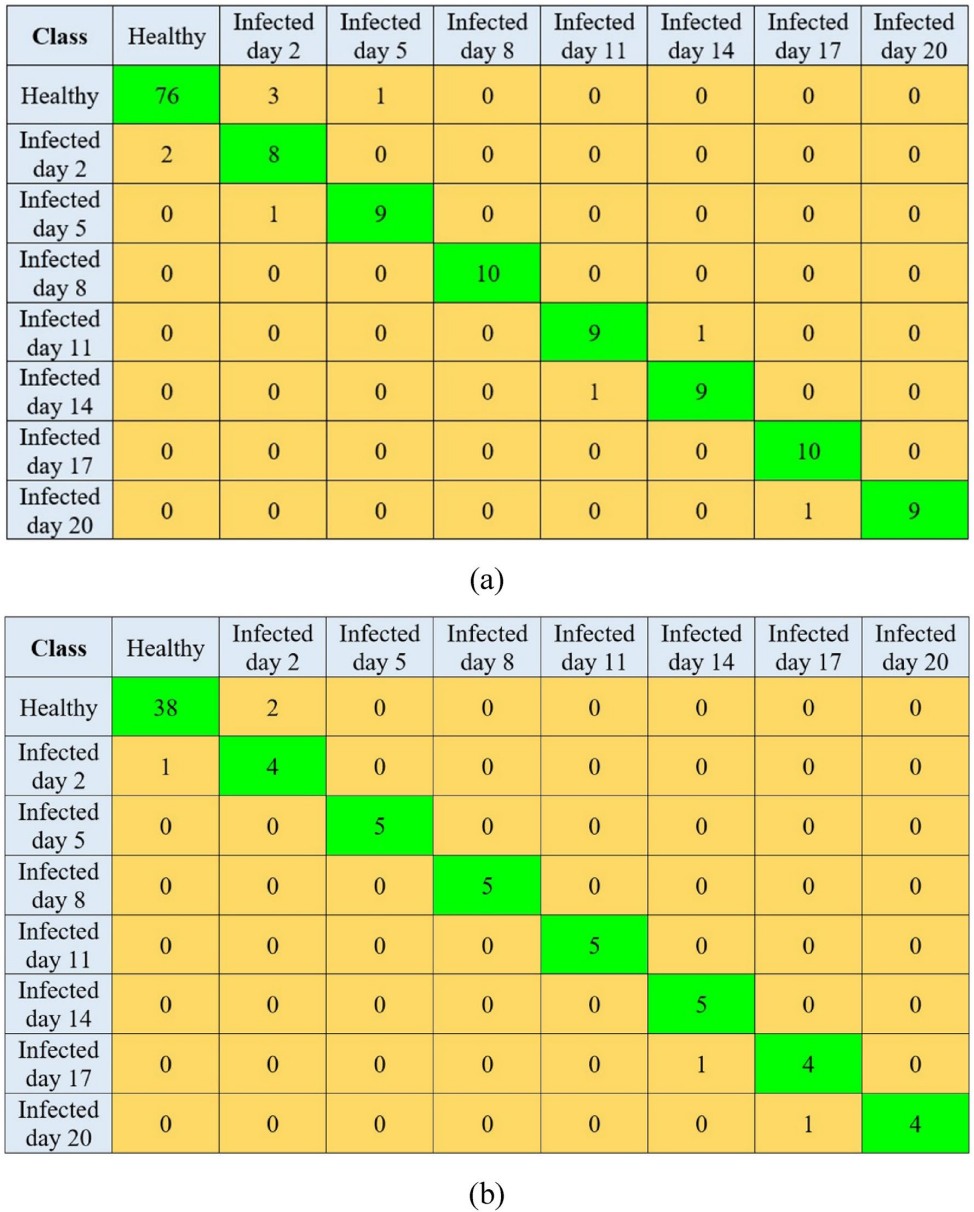
Confusion matrix of SNV-none-LDA for classifying non-contaminated and contaminated kiwifruit samples of different days in cross-validation (**a**) and evaluation (**b**) stages

**Fig. 8:**
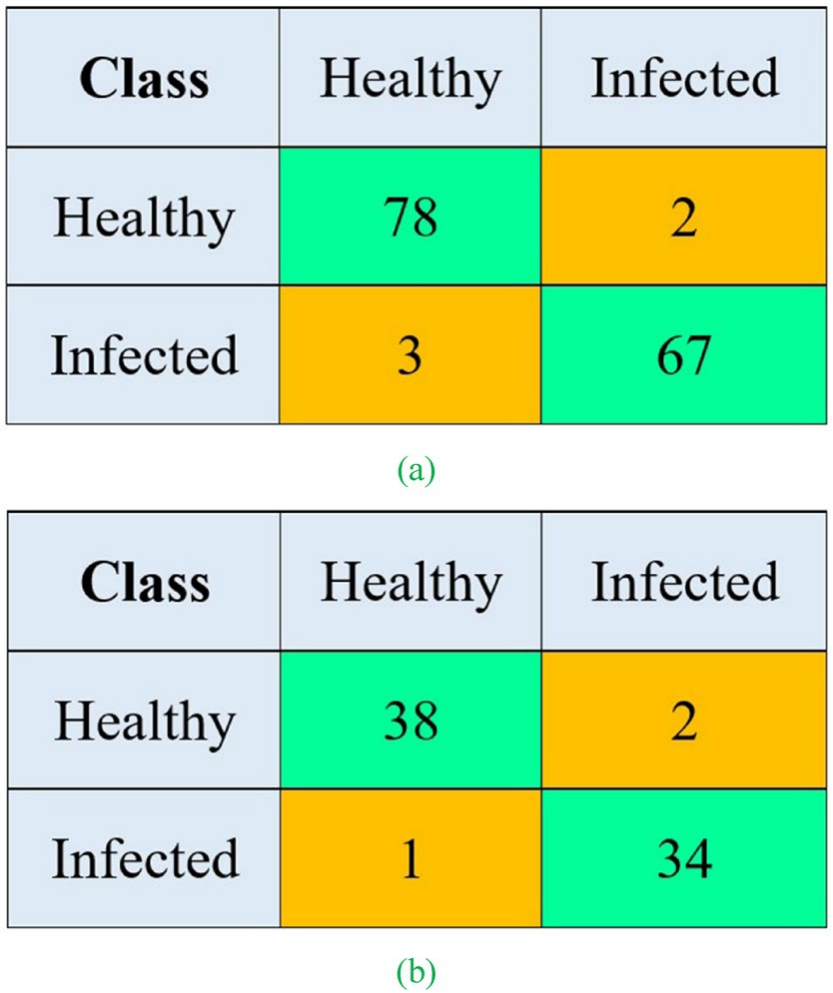
Confusion matrix of SNV-none-LDA for classifying contaminated from non-contaminated kiwifruit samples on calibration (**a**) and evaluation (**b**) datasets

The performance values of LDA and SVM classifiers for discriminating kiwifruit samples on different days after infection using SNV-pretreated spectral features are available in Table 4. Obviously, from Table 4, the LDA classifier with SNV data and no employed feature selection (SNV-none-LDA classifier) resulted in the highest classification performance with the ${accuracy}_{CV}$, ${RMSE}_{CV}$, ${accuracy}_{EV}$, and ${RMSE}_{EV}$ of 93.33%, 0.1286, 93.33%, and 0.1289, respectively. The next rank belonged to the SNV-UVE-LDA model with meaningfully weaker performance than the superior classifier (${accuracy}_{CV}$=90.00%, ${accuracy}_{EV}$=89.33%, ${RMSE}_{CV}$=0.1532, and ${RMSE}_{EV}$=0.1528). This indicated that the information on all spectral reflectance of the kiwifruit samples in the range recorded in this study was necessary to discriminate non-contaminated and diseased kiwifruit samples accurately. The confusion matrices of the SNV-none-LDA classifier on the cross-validation and evaluation are depicted in Fig. 7. Figure 7a shows that 76 out of 80 non-contaminated kiwifruits (95.00% of the samples) were correctly classified. In contrast, 91.43% of the contaminated samples (64 out of 70) were correctly classified. Regarding this high classification rate, the SNV-pretreated data were again fed into the LDA model to classify the infected kiwifruits from the healthy samples. The classification performance was evaluated accordingly. The ${accuracy}_{CV}$, ${accuracy}_{EV}$, ${RMSE}_{CV}$, and ${RMSE}_{EV}$ values of the SNV-LDA model for two-class discrimination were equal to 96.67%, 96.00%, 0.1247, 0.1251, respectively. The corresponding confusion matrices of this classifier in cross-validation and evaluation stages are available in Fig. 8. These results show the high capability of HSI coupled with LDA for classifying non-contaminated kiwifruit samples from gray mold-contaminated ones. Effective application of LDA was also reported in previous HSI-related literature. LDA was successfully employed for the early classification of *Magnaporthe oryzae*-contaminated barley leaves based on the CARS-selected wavelengths with an accuracy of 98% [45]. The LDA model was applied by Sun et al. [72] to detect moldy peanut kernels with an accuracy of 100%. The LDA classifier coupled with the HSI method was also reported to detect sweet potato defects with a total accuracy of 99.52% [73].

**Table 4:** The classification results of kiwifruit samples by LDA and SVM models using SNV spectral data and different wavelength selections

Classifier	Wavelength selection	${accuracy}_{CV}$	${RMSE}_{CV}$	${accuracy}_{EV}$	${RMSE}_{EV}$
**LDA**	None	**93.33**	**0.1286**	**93.33**	**0.1289**
	**CARS**	82.00	0.2065	80.00	0.2076
	SPA	84.67	0.2038	82.67	0.2061
	UVE	90.00	0.1532	89.33	0.1528
SVM	None	80.67	0.2057	77.33	0.2204
	CARS	80.00	0.2074	69.33	0.2487
	SPA	74.67	0.2297	64.00	0.2631
	UVE	76.00	0.2281	64.00	0.2633

Bold text indicates the optimal model structure

**Fig. 9:**
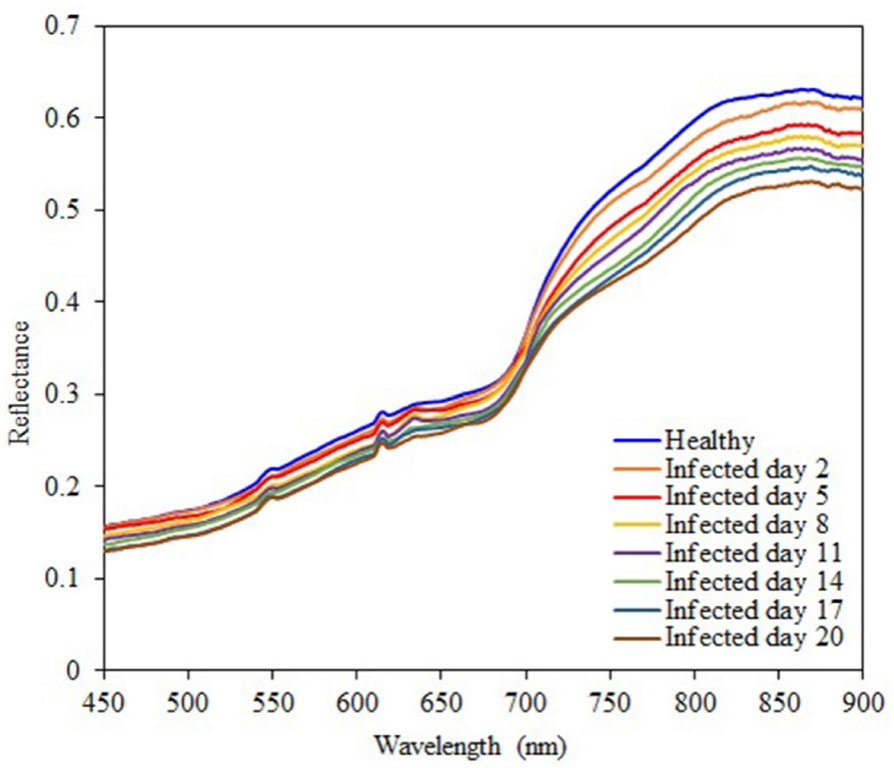
Averages of spectral reflectances of the healthy and infected kiwifruits in the range of 450 nm to 900 nm

**Fig. 10:**
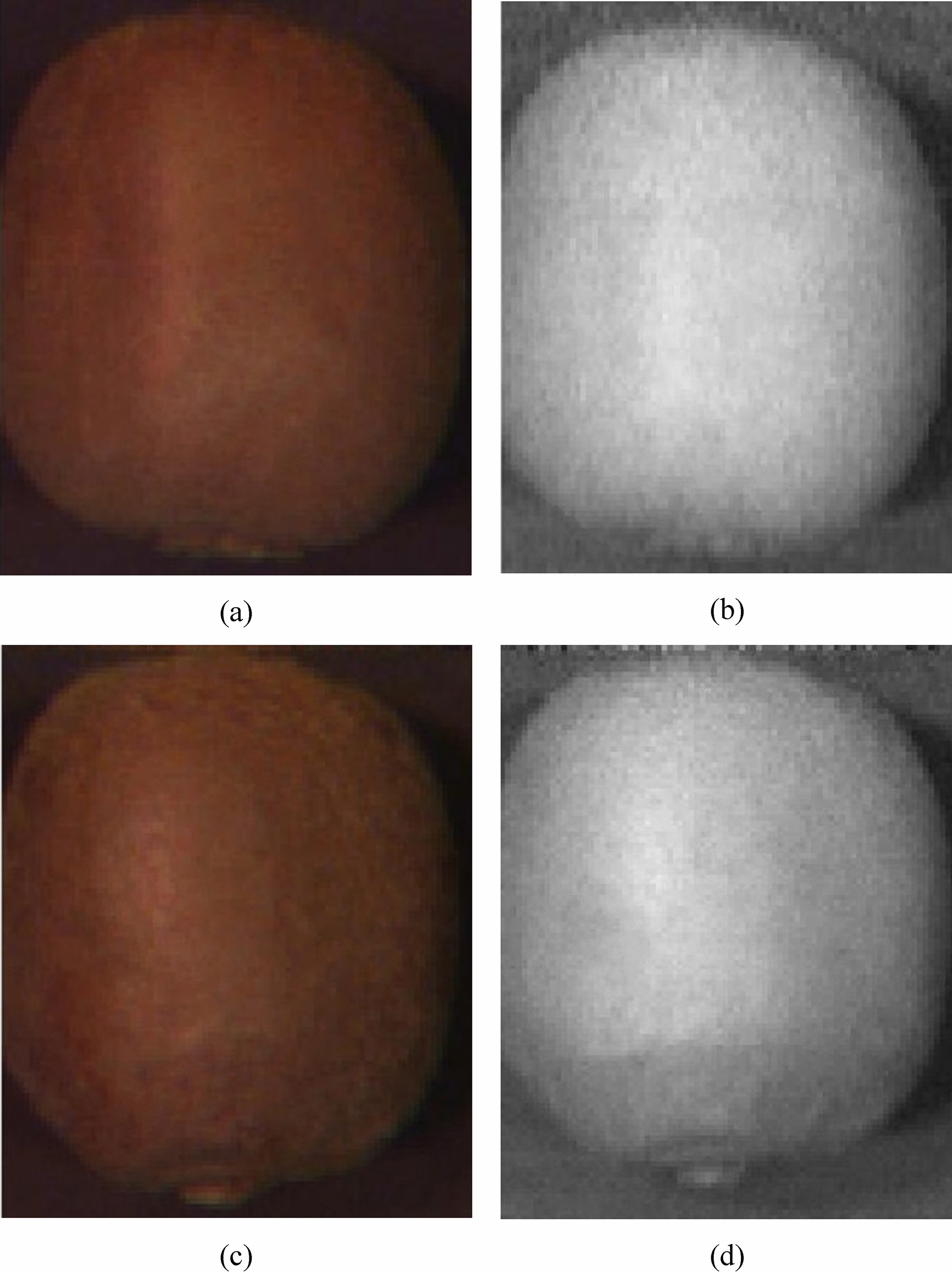
Color images of sample healthy (**a**) and infected (**c**) kiwifruit at day five by overlaying single band images of 685 nm, 546 nm, and 435 nm spectral bands, besides the single-band images of the healthy (**b**) and infected (**d**) kiwifruit in the wavelength of 786 nm

The average spectral reflectances of the surfaces of healthy kiwifruits and the infected kiwifruits of different days are shown if Fig. 9. From this figure, the lower spectral reflectances in the near infrared (NIR) range indicate more days passed after inoculation. The average spectral values of the healthy kiwifruits in the NIR range were higher than those of the infected regions of the inoculated kiwifruits. The spectral differences were less obvious in the visible range as compared with the NIR range. Figure 10 shows pseudo-color images of one healthy sample and one inoculated sample for day five which have been obtained by overlaying three single-band images: 685 nm for the red component, 546 nm for the green component, and 435 nm for the blue component. This procedure was performed according to Yang et al. [74]. After five days from the start of the samples inoculation, it was still difficult to distinguish the healthy fruit (Fig. 10a) from the infected fruit (Fig. 10b) through visible-range color images. However, it is obvious from the extracted single-band images of 835 nm wavelength that the regions around the peduncle of the infected fruit is darkened (Fig. 10d). This shows that the reflectance range of 750 to 900 nm has a high ability to distinguish between the kiwifruits infected with *Botrytis cinerea* fungi and the healthy fruits.

### Prediction of kiwifruit physiochemical attributes

**Fig. 11:**
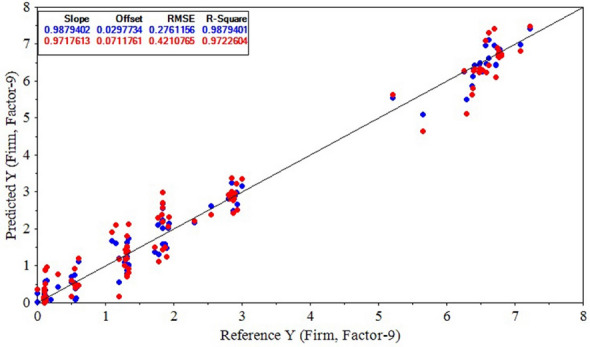
Result of the Savitzky–Golay 1^st^ derivative-CARS-PLSR model for predicting kiwifruit firmness during the experiments

PLSR and SVR models were constructed to predict the variations firmness, SSC, and TA in kiwifruit samples during the experiments by Savitzky–Golay 1^st^ derivative spectral data and different wavelength selection algorithms. The corresponding results are presented in Table 5. According to Table 5, the PLSR model calibrated by CARS-selected wavelengths (Savitzky–Golay 1^st^ derivative-CARS-PLSR model) resulted in the most accurate prediction of kiwifruit firmness. The ${R}_{Cal}^{2}$, ${RMSE}_{Cal}$, ${R}_{CV}^{2}$, and ${RMSE}_{CV}$ criteria of the Savitzky–Golay 1^st^ derivative-CARS-PLSR model were 0.9879, 0.2761, 0.9722, and 0.4211. Figure 11 shows the model predicted vs. experimental (reference) kiwifruit firmness values. The close scatter of dots around the line of identity (black line) delivers the high prediction performance of the Savitzky–Golay 1^st^ derivative-CARS-PLSR model. The Savitzky–Golay 1^st^ derivative-CARS-PLSR model was also evaluated on a separate evaluation dataset (containing 75 samples) and the obtained ${R}_{EV}^{2}$ and ${RMSE}_{EV}$ for firmness prediction were 0.9693 and 0.4331, respectively.

**Table 5:** The prediction results of kiwifruit firmness values by PLSR and SVR models using Savitzky–Golay 1^st^ derivative spectral data and different wavelength selections

Prediction model	Wavelength selection	${R}_{Cal}^{2}$	${RMSE}_{Cal}$	${R}_{CV}^{2}$	${RMSE}_{CV}$
**PLSR**	None	0.9691	0.3739	0.9363	0.6940
	**CARS**	**0.9879**	**0.2761**	**0.9723**	**0.4211**
	SPA	0.9164	0.7266	0.8595	0.9416
	UVE	0.8985	0.8038	0.8709	0.9109
SVR	None	0.9532	0.4318	0.9105	0.7687
	CARS	0.9698	0.3723	0.9616	0.4868
	SPA	0.9171	0.7245	0.8753	0.8852
	UVE	0.9218	0.7009	0.8583	0.8947

Bold text indicates the optimal model structure

**Fig. 12:**
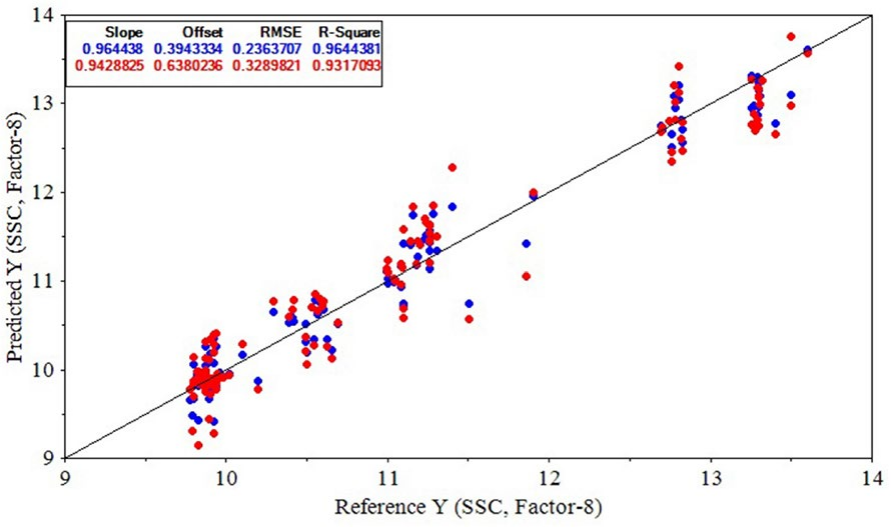
Result of the Savitzky–Golay 1^st^ derivative-CARS-PLSR model for predicting kiwifruit SSC during the experiments

Table 6 shows the performance criteria of PLSR and SVR models for predicting the variations of kiwifruit SSC based on Savitzky–Golay 1^st^ derivative data. It can be observed that the Savitzky–Golay 1^st^ derivative-CARS-PLSR model was the most successful SSC predictor during the experiment. The ${R}_{Cal}^{2}$ and ${RMSE}_{Cal}$ values of this model in the calibration stage were 0.9644 and 0.2364, respectively. The corresponding criteria of ${R}_{CV}^{2}$ and ${RMSE}_{CV}$ in the model evaluation stage were 0.9317 and 0.3290, respectively. Also, Table 6 shows that, in a general view, the CARS-selected wavelengths were the most appropriate features to develop higher accuracy models. Subsequently, the result of the Savitzky–Golay 1^st^ derivative-CARS-PLSR model for predicting SSC based on spectral data is depicted in Fig. 12, showing the high prediction performance of the model. The ${R}_{EV}^{2}$ and ${RMSE}_{EV}$ of Savitzky–Golay 1^st^ derivative-CARS-PLSR model in the evaluation stage were 0.9305 and 0.3311, respectively.

**Table 6:** The prediction results of kiwifruit SSC values by PLSR and SVR models using Savitzky–Golay 1^st^ derivative spectral data and different wavelength selections

Prediction model	Wavelength selection	${R}_{Cal}^{2}$	${RMSE}_{Cal}$	${R}_{CV}^{2}$	${RMSE}_{CV}$
**PLSR**	None	0.9564	0.2645	0.7800	0.5903
	**CARS**	**0.9644**	**0.2364**	**0.9317**	**0.3290**
	SPA	0.6383	0.7581	0.6007	0.7864
	UVE	0.7981	0.5702	0.7456	0.6308
SVR	None	0.9152	0.3649	0.7548	0.6257
	CARS	0.9514	0.2709	0.9170	0.3651
	SPA	0.6378	0.7558	0.5641	0.8309
	UVE	0.8539	0.4848	0.7137	0.6582

Bold text indicates the optimal model structure

**Fig. 13:**
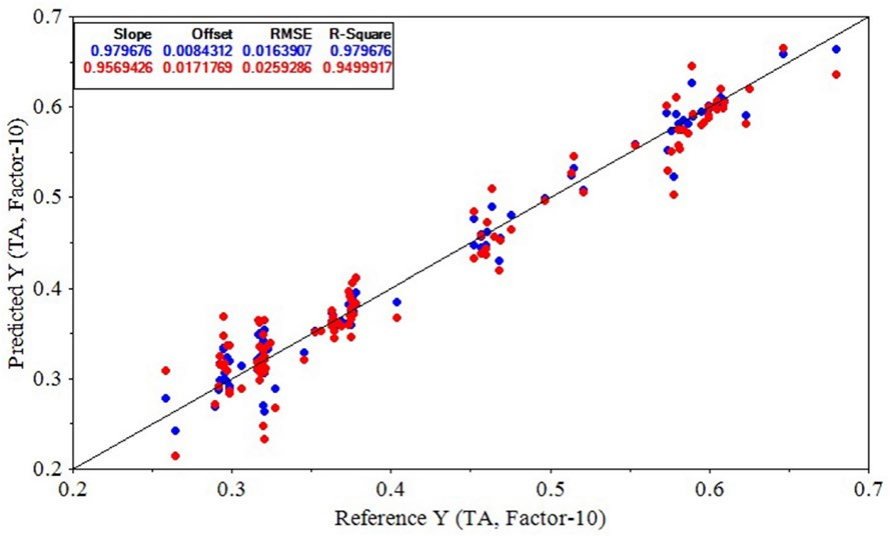
Result of the Savitzky–Golay 1^st^ derivative-CARS-PLSR model for predicting kiwifruit TA during the experiments

**Fig. 14:**
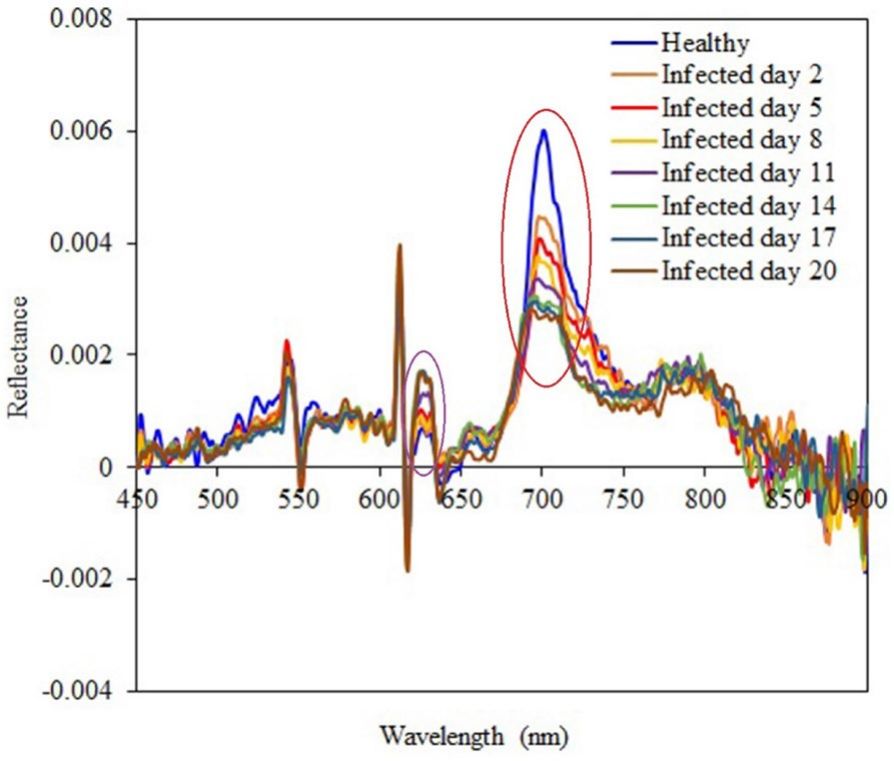
Averages of Savitzky–Golay 1^st^ derivative of spectral reflectances of the healthy and infected kiwifruits in the range of 450 nm to 900 nm

The performance statistics of PLSR and SVR models for predicting TA values based on the Savitzky–Golay 1^st^ derivative spectral data are presented in Table 7. It was observed again that the PLSR model exhibited a more outstanding performance over the SVR model with the ${R}_{Cal}^{2}$ of 0.9797 and ${RMSE}_{Cal}$ of 0.0164 on the model calibration dataset. The Savitzky–Golay 1^st^ derivative-CARS-PLSR model obtained a ${R}_{CV}^{2}$ of 0.9500 and a ${RMSE}_{CV}$ of 0.0259 when it was evaluated in the cross-validation. A graphical representation of the result of the Savitzky–Golay 1^st^ derivative-CARS-PLSR modeling structure for TA prediction is illustrated in Fig. 13, proving this model's high prediction capability. Eventually, the ${R}_{EV}^{2}$ and ${RMSE}_{EV}$ of this model obtained were 0.9543 and 0.0251, respectively, for TA prediction. These crtitera demonstrate the robustness of Savitzky–Golay 1^st^ derivative-based model for monitoring the physiochemical indices of kiwifruits. Figure 14 shows the average Savitzky–Golay 1^st^ derivatives of spectral reflectances from the healthy and infected fruits. Although the general variation trend of the derivative graphs of the different samples are similar to each other, it is clear that in two wavelength ranges of 685–715 nm and 620–635 nm (marked in the Fig. 14 by red and pink elliptic respectively), there is a visible difference between the spectral rates. This point can be used to predict the physiochemical characteristics of kiwifruit samples. The infected fruits of later days had almost lower reflectance derivative values in the range of 685–715 nm, while there was an opposite trend in the wavelength range of 620–635 nm. Another point is that there was no obvious difference between the defected samples in the two mentioned wavelength bands from the day 14 to the day 20. This can be related to the small changes in the physiochemical characteristics of the fruits during this period.

**Table 7:** The prediction results of kiwifruit TA values by PLSR and SVR models using Savitzky–Golay 1^st^ derivative spectral data and different wavelength selections

Prediction model	Wavelength selection	${R}_{Cal}^{2}$	${RMSE}_{Cal}$	${R}_{CV}^{2}$	${RMSE}_{CV}$
**PLSR**	None	0.9619	0.0225	0.8183	0.0515
	**CARS**	**0.9797**	**0.0164**	**0.9500**	**0.0259**
	SPA	0.5800	0.0726	0.5714	0.0720
	UVE	0.8072	0.0497	0.7761	0.0537
SVR	None	0.9608	0.0231	0.7982	0.0561
	CARS	0.9642	0.0218	0.9771	0.0317
	SPA	0.5847	0.0745	0.5750	0.0742
	UVE	0.8570	0.0437	0.7812	0.0587

Bold text indicates the optimal model structure

In brief, the results indicated that the Savitzky–Golay 1^st^ derivative-CARS-PLSR arrangement was the most successful predictor of the quality attributes of the kiwifruit samples during moldy gray infection based on hyperspectral data with the R^2^ of more than 0.96 and 0.92 on the calibration and prediction datasets, respectively. Although there is no previous study on detecting and tracking the fungal diseases in kiwifruit using hyperspectral data, the integration of PLSR and HSI was reported to be effective in assisting the quality attributes of fruits. The CARS-selected wavelengths and the PLSR model were used by Xu et al. [75] to predict the SSC and hardness values of Red Sun kiwifruit during freezer storage with an R^2^ of up to 0.88 and 0.89, respectively. The R^2^ of the PLSR model was reported to be 0.94 and 0.92 for predicting Hayward kiwifruit’s SSC and firmness measures during ripening based on the hyperspectral images [29]. Other papers have also reported the successful applications of different wavelength ranges of hyperspectral data for predicting the internal quality of kiwifruits [76–78]. The results of this study conform to the literature reported the application of HSI and machine learning for early detection of the defection and monitoring of the quality parameters of decayed fruits such as honey peaches [79], strawberries [36], pear [80], and blueberries [81]. Considering the high model performances obtained in this research, combining HSI and machine learning techniques can be a promising approach to building an accurate, nondestructive, fast system for detecting and tracking gray mold infection in kiwifruit.

## Conclusion

Application of hyperspectral imaging and chemometrics strategies was investigated for early-stage detecting *Botrytis cinerea* infection during postharvest storage of Hayward kiwifruit. Different spectral wavelength preprocessing algorithms followed by several wavelength selection methods were applied to prepare appropriate input data for classifying non-contaminated and contaminated kiwifruit samples and predicting the variation of kiwifruit physiochemical attributes during the experiments. It was observed that the Savitzky–Golay 1^st^ derivative method effectively removed the scattering interference and background noise. Moreover, the CARS-selected wavelengths were the most optimum features for predicting the variations of kiwifruit quality attributes during the experiments. Eventually, the Savitzky–Golay 1^st^ derivative-CARS-PLSR model structure resulted in the highest prediction performances for monitoring the kiwifruit firmness, SSC, and TA variations, with the R^2^ of 0.9722, 0.9317, 0.9500, respectively, in the cross-validation. Furthermore, the LDA was able to classify contaminated and non-contaminated kiwifruit samples based on SNV-filtered spectra with an accuracy of 96.67%. The results of this study proved the great application potential of hyperspectral imaging and chemometric approaches for detecting the *Botrytis cinerea* infection in kiwifruit and monitoring the variations that occurred in kiwifruit physiochemical attributes due to the infection. This study provides rich scientific support for developing an accurate and nondestructive system for early-stage detecting of gray mold contamination in kiwifruit during postharvest storage.

## Data Availability

Data will be made available from the corresponding author on reasonable request.
